# Resisted Sled Sprint Kinematics: The Acute Effect of Load and Sporting Population

**DOI:** 10.3390/sports9100137

**Published:** 2021-09-30

**Authors:** Katja M. Osterwald, David T. Kelly, Thomas M. Comyns, Ciarán Ó Catháin

**Affiliations:** 1 Department of Sport and Health Sciences, Athlone Institute of Technology, N37 HD68 Athlone, Ireland; davidkelly@ait.ie (D.T.K.); ciaranocathain@ait.ie (C.Ó.C.); 2 SHE Research Group, Athlone Institute of Technology, N37 HD68 Athlone, Ireland; 3 Department of Physical Education and Sport Sciences, University of Limerick, V94 T9PX Limerick, Ireland; Tom.Comyns@ul.ie; 4 Health Research Institute, University of Limerick, V94 T9PX Limerick, Ireland

**Keywords:** resisted sprints, sled sprint, kinematics, gait, team sport, sprint athlete

## Abstract

In this study, we assessed the acute kinematic effects of different sled load conditions (unloaded and at 10%, 20%, 30% decrement from maximum velocity (Vdec)) in different sporting populations. It is well-known that an athlete’s kinematics change with increasing sled load. However, to our knowledge, the relationship between the different loads in resisted sled sprinting (RSS) and kinematic characteristics is unknown. Thirty-three athletes (sprinters n = 10; team sport athletes n = 23) performed a familiarization session (day 1), and 12 sprints at different loads (day 2) over a distance of 40 m. Sprint time and average velocity were measured. Sagittal-plane high-speed video data was recorded for early acceleration and maximum velocity phase and joint angles computed. Loading introduced significant changes to hip, knee, ankle, and trunk angle for touch-down and toe-off for the acceleration and maximum velocity phase (*p* < 0.05). Knee, hip, and ankle angles became more flexed with increasing load for all groups and trunk lean increased linearly with increasing loading conditions. The results of this study provide coaches with important information that may influence how RSS is employed as a training tool to improve sprint performance for acceleration and maximal velocity running and that prescription may not change based on sporting population, as there were only minimal differences observed between groups. The trunk lean increase was related to the heavy loads and appeared to prevent athletes to reach mechanics that were truly reflective of maximum velocity sprinting. Lighter loads seem to be more adequate to not provoke changes in maxV kinematics. However, heavy loading extended the distance over which it is possible to train acceleration.

## 1. Introduction

Sprinting is a powerful action where the muscles of the lower limbs produce high amounts of vertical and horizontal net force with each step [1]. Research indicates that the body is oriented with a large degree of forward lean during the acceleration phase but becomes more upright as velocity increases and as athletes progress through a sprint [2,3]. Sprint performance (SP) is a result of both the absolute physical capability of the body, and technical ability to apply this raw capacity in an effective manner [4,5]. Recent literature has established that acceleration and maximal velocity SP are related to the technical ability to apply resultant ground reaction forces in a more horizontal direction [6]. Thus, faster athletes have a constant forward orientation, not only through acceleration but also in the maximum velocity phase [1,6].

When attempting to improve full SP, an increase in the ability to produce force and power (physical), and/or improved technical execution is targeted by coaches [7]. Resistance training is a way of improving muscular power [8,9,10,11] and exercises such as squats, power cleans and deadlifts comprise the base of most strength and conditioning programs for athletes to develop speed and power. Although these movements may reflect specificity from a physical development perspective, they lack movement similarity. Despite this, movements such as squats have been shown to have positive effects on sprint performance (2.3%); however, they appear to benefit actions such as vertical jumps that are more kinematically similar, to a greater extent (21%) [12]. Therefore, when training to improve physical capacity, it appears that actions that display greater movement similarity may transfer this improved physical capacity to a greater extent (3.3–9.1%). It may be logical to assume that the addition of external load during a sprint may more closely mimic the action of sprinting while targeting increased force and power output due to the additional resistance. While resisted sprint training (RST) targets the development of force and power output generally, recent interventions use a more targeted approach to improve specific phases of the sprint at specific velocities [13]. As a result, resisted sled sprinting (RSS) has become a common sprint training method utilized by many sports teams and athletes, and its popularity is reflected in its inclusion in recent publications [7,14,15,16,17,18,19,20,21,22,23,24,25,26,27,28,29,30,31,32,33,34,35,36,37,38]. In addition, multiple systematic reviews have demonstrated positive effects of RST on full SP across multiple loading conditions (5–80%BM) [7,15]. More specifically, RST appears to improve acceleration [7] (*p* = 0.0001; effect size (ES) 0.61) [15], but not maximum velocity performance (*p* = 0.25; ES 0.27) [15]. With more recent literature demonstrating benefits of very heavy loads for acceleration and maximum velocity performance (50% and 60%Vdec) [39].

Although RST appears to be an effective training modality for improving sprint performance, to date there remains a lack of clarity around how loading influences kinematics during RST both acutely and following training interventions [7,37,40]. A number of studies have assessed the acute kinematics of RST and demonstrated that loading (10–40% BM) results in decreased step length, swing phase duration, step frequency, but increased contact time (CT), trunk lean [38] and knee flexion relative to unloaded sprinting [33,40,41]. Despite these observations of acute kinematic changes relative to unloaded sprinting, research indicates that these do not appear to transfer into unloaded sprinting and that RST is still an effective modality for improving sprint performance. Alcaraz, et al. [39] and Lahti, et al. [38] assessed longitudinal effects of RST and reported no significant changes in CT and joint kinematics [38] across different phases of the sprint after a 4-week intervention with trained athletes (mostly sprinters, load of 7.5%Vdec) and a 9-week training intervention in field sport athletes (50% and 60%Vdec) [38]. However, Lahti, et al. [38] only assessed trunk lean and hip angle and did not examine any other lower body joint angles.

Moreover, across the literature, there is a lack of standardisation of resistance protocols. Most current research on acute changes in kinematics to date has used %BM [15,18,22,24,34,42,43,44,45,46], as well as systematic reviews on interventions [7,15]. Working at a given %BM can lead to a large variability between athletes in the amount they are slowed down during RSS. Alternatively, current research used load that is causing a reduction in maximum velocity (Vdec) when compared to unresisted sprinting [31,38,47,48]. This makes comparison of research very challenging.

Furthermore, no studies [14,17,18,20,22,29,30,34,37,38,40,41,49,50] have assessed multiple loads on multiple joint angles for acceleration and maximum velocity phase or compared different sporting populations, and it is therefore unclear if athletes from different sports with varying physiological characteristics display similar kinematics when completing RST at different loads. For example, it is plausible that sports that place a larger training emphasis on sprinting (sprinters vs. team sport athletes) may provide athletes with a greater ability to complete RST under heavier loads, without negatively impacting sprint kinematics. As a result, it is possible that athletes with smaller kinematic differences may see a larger transfer effect. However, this is currently unknown as the acute changes during RSS measured pre intervention have not been reassessed after intervention. This study is a first step towards understanding these differences.

With this in mind, the aim of this study was to examine the kinematic characteristics of RSS under different loading conditions and compare how these loads influence kinematics in sprint athletes and invasion team sport athletes.

The results of this study will provide coaches with important information that may influence how RSS is employed as a training tool to improve full SP for acceleration and maximal velocity running and how prescription may change based on sporting population.

## 2. Materials and Methods

### 2.1. Participants

Thirty-three healthy participants (sprint (10) team sport (23), 21.4 ± 3.3 years, 185.8 ± 8.2 m, 85.2 ± 11.8 kg) volunteered and provided written informed consent. Participants were recruited if they (a) had experience with resistance and sprint training (minimum of 18 months), (b) were currently strength training, (c) were currently participating in competitive sprinting or team sport and (d) were injury free for a minimum for 6 months. These criteria were chosen in order to reduce the chances of a possible injury and to prevent delayed onset muscle soreness which might be caused by the dynamic nature of the testing protocols, as well as to improve ecological validity. The study was approved by the Athlone Institute of Technology Ethics Committee (approval code: 20180206), and all procedures were completed in accordance with the declaration of Helsinki.

### 2.2. Experimental Approach to the Problem

This study assessed the kinematics of acceleration and maxV phases of sprint and team sport athletes during RSS at multiple loads (0, 10, 20, and 30%Vdec) using a between-within repeated measures design. Athletes completed 2 testing days that included a familiarization day and an experimental day, which were separated by a minimum of 48 h. On both days participants completed 40 m sprints (12 each) on an indoor running track at each of the above listed loading conditions. Kinematics were only assessed during experimental measures.

### 2.3. Procedures

**Figure 1:**
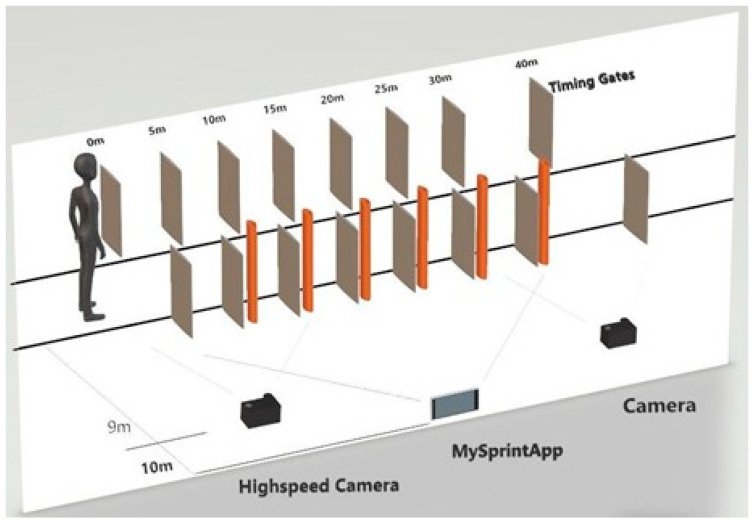
Experimental Set-up.

The following set-up was employed during both familiarization and experimental trials. Timing gates (Brower Timing Systems, Draper, UT USA) were placed at 5 m intervals over a 40 m distance on an indoor running track (Mondo, Sportflex Super X 720 K39, Alba, Italy). This can be observed in Figure 1. For resisted runs, a weighted sled was attached to each participant by a 3.6-m cord and waist harness to minimize lateral displacements during sprinting [42]. Prior to the commencement of trials participants completed a standardized 15-min warm-up using the RAMP protocol [51], and finished with sprints that increased in intensity, as in Jeffreys [51]. Participants were then provided with a further 5-min to complete additional self-selected warm-up exercises.

Familiarization: Participants performed three 40 m sprints at each loading condition (unloaded, 10%, 20% and 30%Vdec) in a randomized order. A minimum 5-min rest period was provided in between each sprint [52]. The method for calculating the load-velocity relationship established by Lockie, et al. [30] was employed to estimate loading during familiarization trials. However, data generated from these trials was then used to adjust loadings by creating an individual linear regression equation for each participant that indicated the required load to reach the planned Vdec (10%, 20% and 30%Vdec) [53]. Participants wore athletic training shoes (no spikes, boots, or cleats) to ensure the consistency of the measurements when comparing different types of athletes.

Experimental trials: Participants performed three 40 m sprints under each loading condition. A minimum 5-min rest period was provided in between each sprint [54]. Participants conducted 12 sprints in total: 3 with a load of 10%Vdec, 3 with a load of 20%Vdec, 3 with a load of 30%Vdec and 3 unresisted sprints. The athletes started with unresisted sprints and then completed the remaining loads in a randomized order. In addition to the set-up described above, sprints during experimental trials were recorded for examination on two different high-speed cameras (HSC). The experimental set-up can be seen below in Figure 1. The HSC were placed at nine meters from the middle of the athlete’s lane and the optical axis of the HSC was perpendicular to the direction of running. The HSC (Sony RX10 III, iPhone 7) were set at a height of 0.85 m and mounted on a rigid tripod, and the frame rate was set at 250Hz for the HSC and 240Hz for the iPhone 7 [55,56]. Each of the 2 cameras had a field of view of 5 m. The first camera captured the first 5 m (0–5 m), which was considered as the early acceleration phase and the second camera captured 5 m between 25 and 30 m, which was considered as the maximum velocity phase [34,57]. To make video analysis easier, markers (zinc oxide tape) were placed on the right-hand side of the participants’ body. Landmarks were established through palpation and exact locations can be seen in Table 1below. A meter stick was placed in the field of view of each camera, for scaling purposes [58].

**Figure 2:**
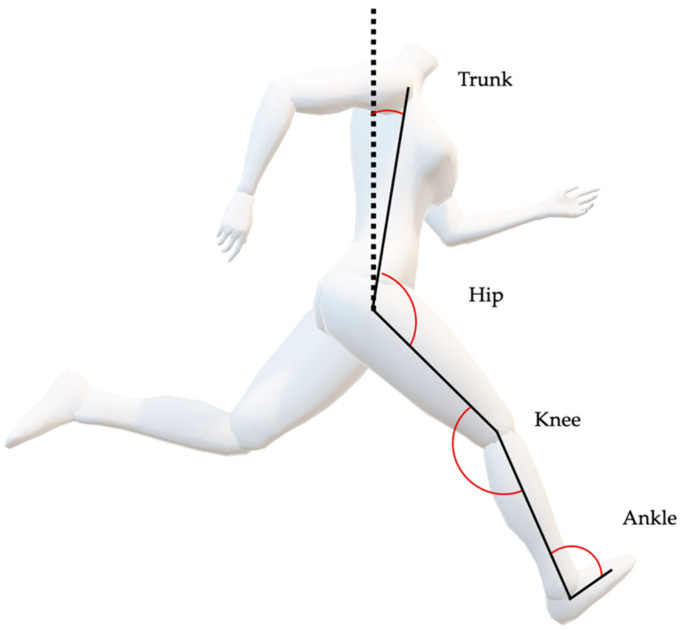
Marker placement. Used to define segments and simplify Dartfish analysis and joint angle definition in the sagittal plane. Hip angle is zero when thigh and trunk are aligned vertically. When the hip angle is positive it is the action of flexion, when it is negative it is the action of extension. When the knee angle gets greater (increases) the knee is extending. When the angle gets smaller (decrease) the knee is flexing. (A decrease in angle refers to the angle becoming smaller and an increase as becoming bigger.) When the ankle angle gets greater the foot is plantarflexing and when it gets smaller the foot is dorsiflexing.

High-Speed-Video Analysis: The video footage collected from the 2 HSC was captured, and a kinematic analysis was completed with Dartfish Software (Fribourg, Switzerland). The tools incorporated into Dartfish high speed video analysis software facilitate the slowing down and magnification of video images in order to calculate joint angles. Joint (trunk, hip, knee, and ankle) angle variables were calculated for the first two contacts of the right foot during the acceleration phase and one (first right foot contact) during the maximum velocity phase of each trial [59]. One step for the maxV phase was deemed sufficient, as kinematics are more consistent due to the athlete sprinting at constant velocity [2]. All angles were measured at toe-off (TO), the first frame in the video where the foot had left the ground and touch-down (TD), the first frame in the video where the foot had contact with the ground [30]. TO and TD were selected as a reflection of what is happening during the force producing component of each stride. Ground contact time is defined as the time between initial ground contact and toe-off and in Dartfish the time of the event of TO was subtracted from the time of the event of TD to calculate CT. Range of motion (ROM) for all loading conditions was calculated from the angles measured at TD and TO as follows. $\mathrm{Percentage}\;\mathrm{Change}\;=\frac{\left(\mathrm{TO}-\mathrm{TD}\right)}{\left|\mathrm{TD}\right|}\times 100$. Percentage change equals the change in value (TO−TD) divided by the absolute value of the original value (TD), multiplied by 100. Joint angle definitions in the sagittal plane are shown in Figure 2.

### 2.4. Statistical Analysis

All data are reported as mean values with standard deviation. Normality of data was determined using the Shapiro–Wilk test. Multiple between-within mixed-model ANOVAs were performed to examine differences for joint angles, CTs and range of motion between groups (field sport athletes vs. sprint athletes) and within groups (0%Vdec, 10%Vdec, 20%Vdec and 30%Vdec).

Mauchley’s test was used to examine sphericity. In cases where the assumption of sphericity was violated, a Greenhouse-Geisser correction was employed. Homogeneity of variance was examined using Levene’s test. Post hoc testing using Bonferroni correction was used to identify where differences lay. Effect size values, partial eta squared (η2p), were also calculated. Threshold values for ES statistics were: Small: 0.2–0.59, Moderate: 0.60–1.19, Large 1.19 [60].The level of significance was set at as *p* = 0.05. The mean of each sprint was used for kinematic variables. Statistical calculations were performed using IBM SPSS 20.0 (Chicago, IL, USA) and MATLAB (R2018a, MathWorks, MA, USA). Intra-tester and inter-trial (between sprints) reliability for kinematic variables was assessed by intraclass correlation coefficient (ICC), coefficient of variation (CV%), and typical error (TE) with 95% confidence intervals, using Hopkins’ spreadsheet [61].

## 3. Results

All results for CT and joint angles for early acceleration and maxV can be found in Table 2 and Table 3. The Shapiro–Wilk test revealed that all data was normally distributed for the acceleration and maximum velocity phases. No significant group*load interactions were identified for any variables, and therefore only main effects for load and group are reported below.

### 3.1. Reliability

For within session (between sprints), ICC with 95% confidence intervals and CV% showed excellent reliability for all kinematic variables (0.96–1.00, CV%: 1.78–3.39). Intra-tester reliability (the same sprint was analyzed twice) also displayed excellent reliability for all variables (0.96–1.00, CV%: 0.63–2.99).

### 3.2. Contact Times

Contact time displayed no difference between groups for both the acceleration and maximum velocity phase (*p* > 0.05). However, there was a significant main effect of load during the acceleration phase for step 1 and 2 (F(3, 84) = 28.54, *p* = 0.00, ηp2 = 0.50); (F(3, 84) = 74.93, *p* = 0.00, ηp2 = 0.72), and during maximum velocity (F(3, 63) = 9.22, *p* = 0.00, ηp2 = 0.27). Post hoc analysis indicated differences between 0% and 10%Vdec, 0% and 20%Vdec and 0% and 30%Vdec (average increase step 1, 0.019–0.046s, *p* = 0.02, 95% CI [−0.03 to −0.01], *p* = 0.00, 95% CI [−0.05 to −0.02] and *p* = 0.00, 95% CI [−0.06 to −0.03]; average increase step 2 0.016–0.046s, *p* = 0.00, 95% CI [−0.02 to −0.01], *p* = 0.00, 95% CI [−0.03 to −0.02] and *p* = 0.00, 95% CI [−0.05 to −0.03]) during the acceleration phase, and between 0% and 30%Vdec during maximum velocity (average increase 0.039s, *p* = 0.01, 95% CI [−0.07 to −0.00]) Table 2.

### 3.3. Joint Angle

There was no significant main effect of group for any joint angles examined (Table 3).

Acceleration Phase Step 1: Increased load resulted in an increase in knee flexion, with differences occurring between 0% and 10%Vdec (average decrease 3.8 degrees, *p* = 0.01, 95% CI [0.48 to 7.24]), between 0% and 20%Vdec (average decrease 7.1 degrees, *p* = 0.00, 95% CI [4.05 to 10.33]) and between 0% and 30%Vdec (average decrease 10.4 degrees, *p* = 0.00, 95% CI [6.15 to 14.76]). No other differences were observed for step 1.

Acceleration Phase Step 2: A similar pattern was displayed for hip angle at TO, with differences observed between 0% and 20%Vdec (decreased by 5.8 degrees, *p* = 0.00, 95% CI [1.39 to 10.26]) and between 0% and 30%Vdec (decreased by 7 degrees, *p* = 0.00, 95% CI [2.23 to 11.87]). Besides hip angle, loading increased knee flexion at TD with differences between 0% and 10%Vdec (average decrease 3.4 degrees, *p* = 0.02, 95% CI [0.29 to 6.52]), between 0% and 20%Vdec (average decrease 7.6 degrees, *p* = 0.00, 95% CI [3.15 to 12.22]), between 0% and 30%Vdec (average decrease 10.7 degrees, *p* = 0.00, 95% CI [6.84 to 14.61]), and between 10% and 20%Vdec (average decrease 4.2 degrees, *p* = 0.01, 95% CI [0.80 to 7.75]). Loading increased ankle dorsiflexion at TD with differences between 0% and 10%Vdec (average decrease 5.3 degrees, *p* = 0.02, 95% CI [0.57 to 10.03]).

Similarly, trunk lean increased at TD and TO with differences observed between 0% and 10%Vdec (average increase 4.3 degrees, *p* = 0.00, 95% CI [−7.82 to −0.81]), between 0% and 20%Vdec (average increase 7.8 degrees, *p* = 0.00, 95% CI [−13.22 to −2.50]) and between 0% and 30%Vdec (average increase 6.7 degrees, *p* = 0.00, 95% CI [−10.39 to −3.10]). Differences occurred at TO for trunk lean between 0% and 10%Vdec (average increase 4.2 degrees, *p* = 0.01, 95% CI [−7.86 to −0.69]), between 0% and 20%Vdec (average increase 6.7 degrees, *p* = 0.00, 95% CI [−9.98 to −3.46]), between 0% and 30%Vdec (average increase 7.2 degrees, *p* = 0.00, 95% CI [−10.10 to −4.38]), and between 10% and 20%Vdec (average increase 2.4 degrees, *p* = 0.03, 95% CI [−4.80 to −0.09]).

MaxV: Statistical analysis revealed a significant main effect of load on trunk lean at TD and TO. Differences at TD occurred between 0% and 20%Vdec (average increase 6 degrees, *p* = 0.03, 95% CI [−11.80 to −0.23]), between 0% and 30%Vdec (average increase 12.4 degrees, *p* = 0.00, 95% CI [−18.74 to −6.68]) and between 10% and 30%Vdec (average increase 10.5 degrees, *p* = 0.00, 95% CI [−16.85 to −4.18]). At TO differences were observed between 0% and 20%Vdec (average increase 9.5 degrees, (*p* = 0.00, 95% CI [−13.66 to −5.48]), between 0% and 30%Vdec (increase 14.4 degrees, *p* = 0.00, 95% CI [−20.11 to −8.73]), between 10% and 20% (average increase 6 degrees, *p* = 0.04, 95% CI [−11.86 to −0.04]), between 10% and 30%Vdec (average increase 10.8 degrees, *p* = 0.00, 95% CI [−18.23 to −3.37]), and between 20% and 30%Vdec (average increase 4.8 degrees, *p* = 0.03, 95% CI [−9.50 to −0.20]).

### 3.4. Range of Motion

Acceleration Phase Step 1: There was a significant main effect of load (F(3, 84) = 6.24, *p* = 0.00, ηp2 = 0.41) and group (F(1, 28) = 9.13, *p* = 0.00, ηp2 = 0.24) for knee ROM, with the sprint group displaying a larger ROM by an average of 10.1%. Furthermore, post hoc analysis revealed a difference in ROM between 0% and 20%Vdec (increase: 4.8%, *p* = 0.03, 95% CI [−9.34 to −0.28]) and 0% and 30%Vdec (increase: 6.8%, *p* = 0.00, 95% CI [−11.84 to −1.86]) for the whole group.

Acceleration Phase Step 2: There was a significant main effect of load for knee ROM (F(3, 84) = 13.98, *p* = 0.00, ηp2 = 0.61) and group (F(1, 28) = 12.05, *p* = 0.00, ηp2 = 0.30), with the sprint group demonstrating a larger ROM by an average of 8.2%. Pairwise comparison revealed differences in ROM between 0% and 10%Vdec (increase: 4.4%, *p* = 0.01, 95% CI [−8.23 to −0.65]), 0% and 20% (increase: 8.4%, *p* = 0.00, 95% CI [−12.56 to −4.24]), 0% and 30%Vdec (increase: 10.9%, *p* = 0.00, 95% CI [−16.05 to −5.78) and similarly, between 10% and 30%Vdec (increase: 6.4%, *p* < 0.05, 95% CI [0.65 to 8.23) for the whole group. In addition, there was a significant main effect of load (F(3, 84) = 4.37, *p* = 0.01, ηp2 = 0.33) but not for group (F(1, 28) = 0.54, *p* = 0.46, ηp2 = 0.02) for ankle ROM. Pairwise comparison revealed a difference in ROM between 0% and 10%Vdec (increase: 9%, *p* = 0.00, 95% CI [-15.94 to -2.10]) only.

MaxV: There was a significant main effect of load (F(3, 63) = 4.37, *p* = 0.00, ηp2 = 0.53) but not group (F(1, 21) = 2.53, *p* = 0.12, ηp2 = 0.10) for knee ROM. Pairwise comparison revealed a difference in ROM between 0% and 30%Vdec (increase: 10.4%, *p* = 0.00, 95% CI [−16.83 to −4.01]). Furthermore, there was a significant main effect of load for ankle ROM (F(3, 63) = 4.59, *p* = 0.01, ηp2 = 0.42) but not group (F(1, 21) = 0.33, *p* = 0.57, ηp2 = 0.02). Pairwise comparison revealed a difference in ROM between 0% and 30%Vdec (increase: 10.8%, *p* = 0.01, 95% CI [−23.80 to 2.10]). No other variables reached significance (*p* > 0.05). During maxV there were no group differences.

Finally, hip ROM was not impacted by any of the loads for both acceleration and maxV phases.

## 4. Discussion

RSS is often prescribed for team sport athletes and sprint athletes [7,20,22,31,42,62] in an effort to improve sprinting performance [31] as it is believed to increase lower-limb power and strength, potentially in a more specific manner than traditional resistance training [7,22,25,49]. Despite this, some concerns with regard to the transfer of RSS training to sprinting performance have been highlighted [15,28,31], due to how RSS may alter kinematics during acceleration and maximum velocity running. However, to date there remains a lack of clarity around what way loading influences kinematics during RSS.

To the authors’ knowledge, this is the first study to investigate the acute effect of multiple loads (0%, 10%, 20% and 30%Vdec) on multiple joint kinematics for different sprint phases and compare how this effect varies in different sporting populations.

Our results confirm that load has a significant effect on kinematics during both acceleration and maximum velocity running and that team sport athletes and sprint athletes, respond to RSS in a very similar manner, with only minor differences between groups.

### 4.1. Contact Time

Contact time is crucial in sprinting as it is the only time an athlete has the ability to create force [63]. RSS has been used to help increase the application of muscular force, especially at the hip, knee, and ankle in trained athletes [30,31,64]. Previous research demonstrates [25,30,65] that CT increases with the addition of load in resisted sprints, with increases of 17–22% reported at loads ranging from 12.6–32.2%BM during acceleration [25,30] and increases of 19 - 26% during maxV with similar ranging loads [25,65]. The current study supports these findings and demonstrated an increase in CT with increasing load; however, this response was not consistent for Acc and maxV (Table 2).

During acceleration CT significantly changed at all loads relative to unloaded (9.3–27.2% increase); however, during maxV, the only significant change occurred between 30%Vdec and unloaded (27.3% increase). The increase in CT during acceleration may be a result of the athlete requiring more time to create momentum and produce force, in order to overcome the higher resistance, and would perhaps be appropriate for the development of hip extension power [31,66]. For example, when squatting at heavier loads research indicates that there is a reduction in movement velocity, increasing the time to produce force, which in turn increases power output at lighter loads [67]. This increase in CT appears consistent across the literature [25,30,42,43,55,64], only a handful of studies have examined the change in CT in unloaded sprinting after an RSS intervention and indicate that this does not appear to transfer to unloaded sprinting [15] and may facilitate a positive adaptation by improving rate of force development (RFD) [38].

### 4.2. Trunk Lean

Our research expands on previous findings [30,68] and indicates that the degree of trunk lean varies with the addition of lighter and heavier loads and can be described as follows: during the acceleration phase there was no change in trunk lean for the initial step, however, trunk angles were significantly greater (greater degree of trunk lean) at all loads at TD and TO during the second step in comparison to unloaded sprinting, with values ranging from 31 degrees in unloaded sprinting to 47 degrees at 30%Vdec. This is in agreement with previous literature [25,30] that has demonstrated an increase in trunk lean across various loading conditions (12.6% BM, to 32.2% BM; 2.5 kg to 10 kg) at TD and TO by 8% to 69%. Higher velocity in the acceleration phase is generated by more forward oriented forces [69] and the greater trunk lean at TD during RSS may help decrease the braking forces associated with landing during acceleration [25,69]. Kunz, et al. [70] investigated the relationship between kinematics and sprinting performance and demonstrated that the forward inclined trunk was an important factor for sprinting performance, as it is a key structure involved in locomotion [71]. Furthermore, the orientation of the maximum force vector strongly correlates with the forward lean of the body at TO (r = 0.93) [69]. Therefore, although the addition of load appears to alter kinematics relative to unloaded sprinting, the increased trunk lean observed, may consequently train athletes to orient their trunk in a position that may facilitate the application of force in a more horizontal direction. However, without a measurement of force we cannot confirm this relationship.

This pattern was also observed during maximum velocity with trunk lean significantly increasing at both 20%Vdec and 30%Vdec at TD, and TO, relative to unloaded sprinting and to 10%Vdec. Therefore, athletes were not achieving an upright running position but remained in a more forward oriented position. This may be problematic during maximum velocity running, as the greater trunk lean associated with the heavier loads may disrupt optimal vertical force application. During maximum velocity running the body should be relatively upright [72], with the overall GRF oriented more vertically, to overcome the effects of gravity and to maintain maximum velocity [2,72,73]. This does not mean that no horizontal force is applied, but vertical forces may play a more important role [73,74]. A recent systematic literature review [15] recommends that there is no optimal load for RST, but that the load should be adapted according to the desired objective. Our findings support existing research [7,22] that recommends from a technical standpoint that lighter loads (<12.5%BM) should be used when implementing RSS methods to train maxV, in order to train the athletes force producing capacity while maintaining maxV mechanics. More specifically, our findings indicate that a load of 10%Vdec allows athletes to maintain mechanics similar to unresisted running, while loads heavier than this may compromise maxV kinematics. On the other hand, using higher loads may extend the distance over which athletes can train acceleration mechanics while using RSS; offering an interesting perspective that may indicate a potential benefit of using heavier loads. However, given the acute nature of the current study, further research is required to assess the extent to which heavy loading may extend the time an athlete spends in acceleration mechanics. It is reported in the literature that very heavy sled loads provide an overload that is efficient in assisting increases in full SP for 5–30 m without violating kinematics for unresisted sprinting [38], but no study measured acute resisted sprinting kinematics pre-post intervention to evaluate if over time kinematics of RSS might improve and become more similar to unresisted sprinting. There exists an interest to see if acute changes have been reduced/eliminated over time. Furthermore, it is unclear if athletes who see less change in kinematics during RSS make more improvements.

### 4.3. Hip Angle

During step 2 of the acceleration phase loads of 20% and 30%Vdec (TO: 170; 169 degrees) resulted in a significant increase in hip flexion relative to unloaded sprinting at TO. There are two possible explanations for the observed reduction in hip extension at TO observed under loaded conditions. Firstly, the athletes might not be strong enough to get through a full ROM with the addition of load [65] and a weakness in the hip abductor muscle typically appears when an athlete is leaning forward with minimal hip extension [75]. It is logical to assume that over time training may allow the athlete to adapt to the additional load, develop stronger hip extensors, and subsequently facilitate hip extension more similar to that observed in unloaded sprinting. However, to our knowledge this has not yet been investigated. Given that hip extension provides the most significant propulsive forces during sprinting [76,77,78], this may offer a positive training adaptation. However, this is unknown and further research is required to determine this.

### 4.4. Knee Angle

During the acceleration phase, knee angles were significantly smaller (less extension) for RSS at all loads at TD in comparison to unloaded sprinting. No significant differences were displayed at TO, with mean knee extension values ranging from 145.4 degrees for unloaded sprinting to 143.4 for 30%Vdec. Knee angle for unloaded sprinting at TO was already close to full extension and similar to previous literature in elite sprinters (142 degrees to 160 degrees) [79]. The findings of this study are in line with previous results from an investigation of RSS [25], even though different loads were used (15%BM and 20%BM). Cronin, et al. [25] reported less extension at TD and no change in extension at TO and suggested that during RSS propulsive forces may act through a greater range, and therefore may comprise a greater proportion of the stance phase. The increase in knee flexion at TD observed with increased load may place the athlete in a position where the shank is in a more horizontal position, potentially allowing athletes to apply force in a more horizontal direction. The ability to apply force more horizontally into the ground is a performance determining factor in acceleration performance [1]. In contrast, Lockie, et al. [30] reported an increase in knee extension (32%BM), with mean values of 156.4 degrees (32%BM) and 148.0 degrees (unloaded) [30]. The authors suggested that this increase in knee extension may indicate that the athlete was attempting to gain an increase in propulsive force through a more vigorous extension of the shank segment [30]. However, these values were measured at maximum extension and not TO. The results of our ROM analysis indicated that athletes went through greater knee ROM when loaded. Increased ROM at the knee may increase the time to develop force and therefore increase impulse during sprinting. Furthermore, sprinters demonstrated greater ROM than team sport athletes. This may indicate that sprint athletes may have stronger hip extensors allowing them to go through a larger ROM or may be more technically proficient. However, this is uncertain as kinetics were not analyzed in the current study and therefore warrants further investigation.

### 4.5. Limitations

As with all investigations, this study should be appreciated considering its limitations. The study sample size was small to moderate, and therefore the findings may not be fully reflective of the population the sample was taken from. The majority of studies including ours look at single time points (TD, TO), however, discrete point analysis may result in loss of important information during other parts of the movement [80,81,82]. A more ideal approach is likely the analysis of waveforms, such as the statistical parametric mapping method, but was beyond the scope of this project [83]. Lastly, due to a limited field of view the measurement of variables during acceleration was only possible for the first two steps. The measurement of variables for example, at the first two steps only, may present a disadvantage, as load-specific changes in kinematics may be present throughout the whole acceleration phase. A step-by-step analysis would elucidate the different phases and changes in kinematics during the sprint [44]. Despite our best attempts at reducing fatigue via appropriate resting periods, it is possible that this still played a role [42]. Sled loads however, were performed in randomized order; therefore, all conditions have been similarly affected by this fact.

## 5. Conclusions

Despite these limitations, this study is novel and has added to the existing body of knowledge, advanced research on RSS and has important practical implications to be considered. This study investigated the effect of RSS on sprint kinematics under various loading conditions similar to previous research; however, the examination of multiple joint angles, across different phases of a sprint, the number of loads and the comparison on how these loads influence kinematics in sprint athletes and invasion team sports athletes is novel.

In conclusion, this study showed that RSS resulted in acute changes in sprint kinematics during sprint acceleration and maxV phases, yet in a distinctive manner when using different loads. Furthermore, this study indicated that both sprint and team sport athletes respond to RSS in a very similar manner. ROM however increased with increasing load to a greater extent for sprint athletes potentially enabling them to create more propulsive forces, which may be due to stronger hip extensors. The utilization of any sled load would appear to ensure that acceleration kinematics at step one were not adversely affected; however, our data indicates that the addition of load alters technique at step two of acceleration and during maxV. Whether or not these changes may adversely affect performance is unclear given the acute nature of the current study. It is possible though that further training under loaded conditions may allow athletes to reach kinematics more similar to unloaded sprinting. It is also possible that the observed change in kinematics, with the addition of load, may positively influence sprinting technique, e.g., a better trunk lean. Furthermore, it is possible that higher level athletes may benefit from more kinematically similar movements or greater levels of specificity than sub elite athletes. Although, the heavier loads did not allow the athletes to reach mechanics that are reflective of maxV, the increase in trunk lean, enabled them to place themselves in an optimal position to maximize propulsive forces, thus, potentially extending the distance over which it is possible to train acceleration. From a practical standpoint, when the main training objective is to improve speed ability without drastically altering kinematics, loads heavier than 10%Vdec may not be appropriate for training maxV. Although we have reported acute kinematic changes, a long-term investigation should include multiple joint angles and a variety of different loads, especially heavy loads, to further investigate the impact on kinematics. This there still is a lack of knowledge in the current literature.

The results of this study provide coaches with important information that may influence how RSS is employed as a training tool and how prescription may change based on sporting population. Practitioners should be aware that load increment during RSS may lead to changes in sprint kinematics, in both acceleration and maxV phases. Although heavy loads provide an overload that is efficient in assisting increases in sprint performance [38] and may be more suitable for optimizing horizontal force production and help athletes to apply force in a position which better reflects the mechanical demands of the sprint, caution is necessary when increasing the load, especially when aiming to replicate unresisted sprint kinematics. For load prescription, it is important for coaches to understand the extent to which RSS can impact kinematics for different sprint phases across different athletic populations, yet still improve sprint performance. To date no study has comprehensively measured kinematic changes across multiple loads and sporting populations.

## Figures and Tables

**Table 1:** Marker placement landmark description [25].

Landmark	Description
Shoulder	Acromion process
Hip	Greater trochanter, located at the proximal, lateral part of the shaft of the femur
Knee	Lateral condyle, at the superior end of the tibia
Ankle	Lateral malleolus, at the low end of the fibula
Toe	Fifth metatarsal bone/transmetatarsal joint at the distal outer edges of the foot (on the shoe)

**Table 2:** Ground contact times between groups.

		First Ground Contact (s)	Second Ground Contact (s)	MaxV Ground Contact (s)
Load	Group	Mean ± SD	Mean ± SD	Mean ± SD
0%	Sprint	0.17 (±0.01)	0.14 (±0.01)	0.11 (±0.01)
	Team	0.20 (±0.02)	0.16 (±0.01)	0.13 (±0.06)
	Total	0.19 (±0.02)	0.15 (±0.01)	0.12 (±0.05)
10%	Sprint	0.19 (±0.01)	0.16 (±0.01)	0.12 (±0.00)
	Team	0.22 (±0.02)	0.17 (±0.01)	0.12 (±0.01)
	Total	0.21 (±0.02) *	0.17 (±0.01) *	0.12 (±0.01)
20%	Sprint	0.22 (±0.03)	0.17 (±0.01)	0.13 (±0.01)
	Team	0.23 (±0.03)	0.18 (±0.02)	0.14 (±0.01)
	Total	0.22 (±0.03) *	0.18 (±0.02) *	0.14 (±0.01)
30%	Sprint	0.22 (±0.02)	0.20 (±0.02)	0.16 (±0.01)
	Team	0.24 (±0.04)	0.20 (±0.02)	0.16 (±0.02)
	Total	0.24 (±0.04) *	0.20 (±0.02) *	0.16 (±0.02) *

* *p* < 0.05 significant difference compared to 0%Vdec.

**Table 3:** Kinematic variables for acceleration phase steps 1 (S1) and 2 (S2) and maxV phase for all athletes.

**Acceleration Phase**								
**Step 1**	**Hip Angle (°)**		**Knee Angle (°)**		**Ankle Angle (°)**		**Trunk Angle (°)**	
	**TD** **(Mean ± SD)**	**TO** **(Mean ± SD)**	**TD** **(Mean ± SD)**	**TO** **(Mean ± SD)**	**TD** **(Mean ± SD)**	**TO** **(Mean ± SD)**	**TD** **(Mean ± SD)**	**TO** **(Mean ± SD)**
0%	101.7 (± 9.46)	177.2 (±7.34)	112.3 (±7.89)	146.7 (±9.55)	102.5 (±8.74)	136.9 (±9.66)	48.2 (±19.34)	45.7 (±19.40)
10%	97.6 (±10.84)	170.9 (±14.09)	108.4 (±8.46) *	144.1 (±21.78)	101.2 (±8.43)	135.2 (±9.10)	51.7 (±14.81)	46.8 (±6.51)
20%	98.4 (±11.58)	171.3 (±7.13)	105.1 (±8.27) *	146.3 (±9.91)	99.1 (±19.75)	136.3 (±9.84)	49.1 (±7.34)	47.8 (±5.90)
30%	99.9 (±11.69)	170.1 (±8.80)	101.8 (±7.39) *	144.7 (±10.29)	98.6 (±19.08)	135.2 (±9.24)	48.6 (±8.15)	46.9 (±5.18)
Load (*p*-value, ES)	*p* = 0.16, ηp2 = 0.05	*p* = 0.14, ηp2 = 0.08	*p* = 0.00, ηp2 = 0.46	*p* = 0.55, ηp2 = 0.01	*p* = 0.72, ηp2 = 0 0.01	*p* = 0.51, ηp2 = 0.02	*p* = 0.62, ηp2 = 0.01	*p* = 0.57, ηp2 = 0.00
Group (*p*-value, ES)	*p* = 0.38, ηp2 = 0.07	*p* = 0.05, ηp2 = 0.04	*p* = 0.54, ηp2 = 0.01	*p* = 0.05, ηp2 = 0.12	*p* = 0.13, ηp2 = 0.08	*p* = 0.75, ηp2 = 0.00	*p* = 0.87, ηp2 = 0.00	*p* = 0.68, ηp2 = 0.00
Step 2								
0%	113.5 (±9.15)	177.0 (±7.34)	121.6 (±6.55)	150.8 (±7.29)	104.9 (±7.80)	132.1 (±6.87)	34.3 (±7.16)	33.0 (±5.90)
10%	108.3 (±10.58)	169.8 (±14.09)	118.2 (±6.73) *	151.6 (±7.58)	99.6 (±7.89) *	133.4 (±6.94)	38.7 (±7.61) *	37.3 (±6.71) *
20%	108.1 (±9.24)	170.8 (±7.13) *	113.4 (±7.87) *^	150.2 (±8.55)	102.9 (±8.18)	135.3 (±6.93)	42.2 (±12.31) *	39.7 (±5.61) *^
30%	107.0 (±10.27)	169.5 (±8.8) *	110.9 (±6.19) *	148.5 (±7.72)	101.6 (±7.43)	132.7 (±6.49)	41.0 (±7.49) *	40.2 (±5.39) *
Load (*p*-value, ES)	*p* = 0.00, ηp2 = 0.18	*p* = 0.00, ηp2 = 0.13	*p* = 0.00, ηp2 = 0.49	*p* = 0.11, ηp2 = 0.06	*p* = 0.01, ηp2 = 0.11	*p* = 0.06, ηp2 = 0.08	*p* = 0.00, ηp2 = 0.27	*p* = 0.00, ηp2 = 0.43
Group (*p*-value, ES)	*p* = 0.28, ηp2 = 0.04	*p* = 0.11, ηp2 = 0.08	*p* = 0.22, ηp2 = 0.05	*p* = 0.05, ηp2 = 0.14	*p* = 0.32, ηp2 = 0.03	*p* = 0.33, ηp2 = 0.03	*p* = 0.29, ηp2 = 0.03	*p* = 0.22, ηp2 = 0.04
**Maximum Velocity Phase**								
	**Hip Angle (°)**		**Knee Angle (°)**		**Ankle Angle (°)**		**Trunk Angle (°)**	
	**TD** **(Mean ± SD)**	**TO** **(Mean ± SD)**	**TD** **(Mean ± SD)**	**TO** **(Mean ± SD)**	**TD** **(Mean ± SD)**	**TO** **(Mean ± SD)**	**TD** **(Mean ± SD)**	**TO** **(Mean ± SD)**
0%	120.3 (±35.68)	133.4 (±88.86)	121.5 (±45.89)	139.1 (±39.63)	83.7 (±51.32)	106.1 (±50.47)	10.6 (±4.87)	9.9 (±5.52)
10%	110.6 (±50.03)	164.9 (±72.75)	138.6 (±39.63)	134.3 (±53.00)	70.4 (52.51)	125.9 (±25.80)	12.8 (±6.76)	13.5 (±6.97)
20%	109.3 (±45.32)	179.9 (±52.07)	128.6 (±35.96)	135.1 (±47.42)	58.5 (±50.39)	117.2 (±41.51)	16.6 (±8.33) *	19.5 (±6.00) *^
30%	99.8 (±51.92)	152.9 (±70.99)	112.2 (±40.53)	147.0 (±29.17)	62.2 (±48.34)	108.7 (±51.63)	23.3 (±9.95) *^	24.3 (±8.66) *^˟
Load (*p*-value, ES)	*p* = 0.57, ηp2 = 0.03	*p* = 0.46, ηp2 = 0.08	*p* = 0.10, ηp2 = 0.09	*p* = 0.70, ηp2 = 0.01	*p* = 0.30, ηp2 = 0.05	*p* = 0.46, ηp2 = 0.03	*p* = 0.00, ηp2 = 0.40	*p* = 0.00, ηp2 = 0.52
Group (*p*-value, ES)	*p* = 0.86, ηp2 = 0.00	*p* = 0.38, ηp2 = 0.39	*p* = 0.14, ηp2 = 0.09	*p* = 0.20, ηp2 = 0.07	*p* = 0.69, ηp2 = 0.00	*p* = 0.33, ηp2 = 0.04	*p* = 0.79, ηp2 = 0.00	*p* = 0.13, ηp2 = 0.10

TO = Toe-off, TD = Touchdown, ηp2: Effect size (Small: 0.2–0.59, Moderate: 0.60–1.19, Large 1.19>), * = *p* < 0.05 significant difference relative to 0%Vdec, ^ = *p* < 0.05 significant difference relative to 10%Vdec, ˟ = *p* < 0.05 significant difference relative to 20%Vdec.

## Data Availability

The data presented in this study are available on reasonable request from the corresponding author.
